# Nutritional status and determinants among primary school students in the challenging terrain of northern mountainous Vietnam

**DOI:** 10.1038/s41598-025-28827-4

**Published:** 2025-12-02

**Authors:** Duong Thuy Thi Truong, Trung Thanh Nguyen, Hoa Thanh Thi Le, Hung Le Xuan

**Affiliations:** 1 Department of Nutrition and Food Safety, University of Medicine and Pharmacy - Thai Nguyen University, Thai Nguyen, Vietnam; 2 Bao Lac Medical Center, Cao Bang, Vietnam; 3 Department of Environmental Health and Occupational Health, University of Medicine and Pharmacy - Thai Nguyen University, Thai Nguyen, Vietnam; 4 Department of Research Methodology and Biostatistics, School of Preventive Medicine and Public Health, Hanoi Medical University, Hanoi, Vietnam

**Keywords:** Malnutrition, Nutritional status, Childhood obesity, Undernutrition, Socioeconomic factors, Vietnam, Nutrition disorders, Nutrition, Risk factors

## Abstract

Malnutrition remains a significant public health challenge among primary school students in the northern mountainous region of Vietnam. We conducted a cross-sectional survey in two primary schools in Trung Khanh District, Cao Bang Province (Trung Khanh Town: *n* = 476; Dam Thuy: *n* = 296). Anthropometry followed WHO protocols, and nutritional status was classified using the WHO 2007 growth reference (5–19 years) based on BMI-for-age Z-scores. A parent/guardian questionnaire captured household sociodemographics, parental nutrition knowledge, children’s dietary habits, and physical activity. In total, 772 students participated. Thinness prevalence was 18.1% (95% CI: 15.4–21.0), and overweight/obesity was 1.2% (95% CI: 0.5–2.3). In multivariable models, lower household income was associated with higher odds of thinness (aOR 0.57, 95% CI 0.36–0.88), while sugary-drink and snack consumption were key correlates of BMI status. Findings highlight the double burden of malnutrition in ethnic-minority settings and point to school- and household-level targets for intervention.

**Supplementary Information:**

The online version contains supplementary material available at 10.1038/s41598-025-28827-4.

## Introduction

Malnutrition, encompassing both undernutrition and overnutrition, remains a pressing public health issue worldwide. This double burden of malnutrition-the coexistence of thinness (BMI-for-age) alongside overweight and obesity-has been increasingly observed in low-resource and geographically disadvantaged populations^1^. The northern mountainous region of Vietnam, characterized by harsh terrain, economic constraints, and limited access to health services, poses unique nutritional challenges for primary school children. In Vietnam, stunting affects 19.6% of children under 5 and 16.8% aged 5–19 nationally, with higher rates among ethnic minorities (e.g., 26.4% stunting in targeted ethnic areas)^2,3^. Ethnic minorities show higher stunting (up to 52% in some groups) and micronutrient deficiencies compared to the Kinh majority^4^. Despite ongoing national efforts to address childhood malnutrition, data on dietary disparities and associated factors in this remote region remain scarce. Studies across Southeast Asia and Asia, such as those conducted in Indonesia (Hidayat et al., 2020) and China (Tang et al., 2020), reveal that low socioeconomic status, maternal education, poor dietary diversity, and food security are key determinants of child malnutrition^5,6^. Research further highlights the role of family food security and parental education in shaping children’s nutritional outcomes. In contrast, urban populations are experiencing a rising trend of childhood obesity, driven by shifts toward energy-dense diets and reduced physical activity^7^.

In Vietnam’s northern mountainous areas, children face both forms of malnutrition, with nutrient deficiencies, poor growth outcomes, and an increasing risk of overweight and obesity. While school-based nutrition education programs have been recognized as an effective strategy for improving food literacy and eating behaviors^8–10^, inadequate resources and limited emphasis on nutrition curricula hinder progress in these remote communities^9^. Furthermore, studies indicate that nutritional status significantly influences children’s cognitive and physical development, affecting motor skills, academic performance, and overall health outcomes^7,11,12^.

Despite these insights, comprehensive studies assessing the double burden of malnutrition among primary school students in northern mountainous Vietnam remain limited. Understanding the nutritional status and its determinants, including dietary intake, socioeconomic factors, and school-based interventions, is crucial for developing targeted policies and intervention programs to improve child nutrition in this vulnerable population. This study aims to bridge the knowledge gap by evaluating undernutrition and overnutrition trends, providing evidence for context-specific strategies to enhance child health and well-being in the region.

## Materials and methods

### Study design

This study employed a cross-sectional descriptive design to assess the nutritional status and associated factors among primary school students in the northern mountainous region of Vietnam. A cross-sectional approach was chosen due to its efficiency in capturing prevalence estimates and identifying key determinants of malnutrition within a defined timeframe. Potential biases, such as recall bias in dietary assessment and measurement errors in anthropometry, were minimized through standardized data collection procedures and trained personnel. Standardized procedures included double measurements for anthropometrics (intra-observer error < 0.5 cm/kg); trained personnel were four nutritionists with > 5 years experience, certified in WHO protocols. Questionnaire pre-testing minimized recall bias.

### Study population

#### The study population included

The study population consisted of primary school students enrolled in two schools in Trung Khanh District, Cao Bang Province (a remote, mountainous area with predominantly ethnic minority populations (e.g., Tay, Nung), limited infrastructure, high poverty rates (per Vietnam’s Decree 07/2021/NĐ-CP)^13^, and challenges in accessing health/nutrition services):

- Trung Khanh Town Primary School (476 students).
- Dam Thuy Primary School (296 students).

In addition, parents or primary caregivers of the students were included to provide relevant demographic and dietary habit information.

#### Inclusion and exclusion criteria

Inclusion Criteria:

- Students actively enrolled in Grades 1–5 at the time of data collection.
- Students who voluntarily participated with written parental/guardian consent.
- Parents/guardians who agreed to provide information through structured questionnaires.

Exclusion Criteria:

- Students diagnosed with acute or chronic illnesses (e.g., tuberculosis, HIV/AIDS, metabolic disorders) that might influence their nutritional status.
- Students who were absent during the study period or whose parents/guardians declined to participate.

### Sample size and sampling method

A total enumeration sampling method was used, including all students from the two selected primary schools (*N* = 772). The study sites were purposefully selected based on feasibility, geographical representation (one urban-adjacent town school, one remote village school), and the willingness of school authorities to facilitate data collection. With total enumeration (*N* = 772), post-hoc power analysis (G*Power software) showed > 80% power to detect malnutrition prevalences of 15–30% (based on national estimates) at α = 0.05. Compared to district/national: Our thinness rate (18.1%) is similar to national ethnic minority estimates (15–20%); overweight/obese (1.2%) is lower than urban national (32.7% in 7–11 year-olds).

Limitation: Not nationally representative.

### Data collection

#### Anthropometric measurements

Anthropometric assessments were conducted following the WHO standard procedures^14^:

- Weight Measurement: Measured using a SECA electronic scale (Japan, precision 0.1 kg). Each student was weighed twice, and the average was recorded.
- Height Measurement: Measured using a UNICEF wooden stadiometer (precision 1 mm). Height was recorded twice, and the average was taken.
- Body Mass Index (BMI): Calculated as weight (kg)/height² (m²). The WHO 2007 growth reference standards were applied for classification using the WHO AnthroPlus software.

To ensure consistency, all measuring instruments were calibrated before data collection, and trained fieldworkers conducted all anthropometric assessments using standardized protocols.

Data collection occurred at schools from January to December 2022. Parents were invited via school notices; the refusal rate was 5% (*n* = 40, mainly due to scheduling).

#### Nutritional status classification

The WHO 2007 Z-score classification system was applied:

- Underweight (Weight-for-Age, W/A): Z-score < −2 SD.
- Stunting (Height-for-Age, H/A): Z-score < −2 SD.
- Wasting (BMI-for-Age, BMI/A): Z-score < −2 SD.
- Normal Nutrition: Z-score between − 2 SD and + 1 SD.
- Overweight: Z-score > + 1 SD.
- Obesity: Z-score > + 2 SD.

#### Questionnaire and survey

A structured questionnaire was administered to parents/guardians to collect sociodemographic and dietary habit data. The questionnaire was developed based on validated nutrition assessment tools (based on FAO Dietary Diversity Score and Vietnamese National Nutrition Survey questionnaires) and pre-tested on a small sample to ensure clarity and reliability (piloted on 30 parents from a nearby district; refined for clarity, e.g., simplified snack questions).

Collected information included:

- Demographics: Age, gender, ethnicity, parental education, occupation, and household income.
- Birth weight: Self-reported by parents/guardians.
- Dietary habits: Frequency of breakfast consumption, intake of fried foods, high-fat foods, sugary snacks, and carbonated beverages.
- Physical activity and sedentary behavior: Daily activity level, screen time, and time spent exercising.
- Parental knowledge and practices related to child nutrition.

Assessment criteria:

- Eating habits:
  - Frequent consumption of processed foods, skipping breakfast, and late-night eating.
  - Regular breakfast consumption: Daily breakfast intake.
  - High sugary drink consumption: ≥ two cans (320 ml each) per week.
- Physical activity:
  - Regular: ≥30 min/day for ≥ 4 days/week.
  - Low: <30 min/day or ≤ 3 Days/week.
- Household economic classification: Based on Vietnam’s multidimensional poverty standard (Decree 07/2021/NĐ-CP)^13^. Households were categorized as poor (below the poverty threshold) or non-poor (average/above the threshold).

Derived Indicators/Variables:

- Meat and Fat Intake: Low (< 3 servings/week), Medium (3–5 servings/week), based on Vietnamese dietary guidelines. Snacks: Processed items like chips, candies, or baked goods (yes: ≥3 times/week).
- Dieting Behavior: Parent-reported restrictive eating (e.g., portion control for health, yes/no).

### Data quality control

To minimize measurement bias, the following quality control measures were implemented:

- Standardized training for all data collectors on anthropometric measurement techniques.
- Regular calibration of scales and stadiometers before each measurement session.
- Double data entry and cross-checking for consistency and accuracy.
- Pilot testing of questionnaires before the primary survey.

### Statistical analysis

Data were entered into EpiData 3.1 and analyzed using SPSS 26.0. Descriptive statistics were calculated for continuous and categorical variables, including mean, standard deviation, and frequency. Associations between categorical variables were assessed using Chi-square (χ²) tests, while T-tests were used to compare means between two groups. Multivariate logistic regression analysis was performed to identify significant predictors of malnutrition; models adjusted for school clustering using generalized estimating equations, and included age (categorized by grade) and school as covariates.

### Ethical considerations

Ethical approval for this study was obtained from the Ethics Committee of the University of Medicine and Pharmacy, Thai Nguyen University (Approval No: 219/ĐHYD-HĐĐĐ on 21 st March 2022). Permission to conduct the study was granted by the Department of Education and Training of Trung Khanh District and the respective school authorities. Informed consent was obtained from all parents or guardians before data collection. To ensure privacy and confidentiality, all data were anonymized and securely stored. Additionally, anthropometric measurements followed WHO protocols, providing standardized and accurate procedures throughout the study. All methods were performed in accordance with the relevant guidelines and regulations.

## Results

### Characteristics of the study population

The sample (*N* = 772) was 50% male, mean age 8.1 ± 1.5 years, predominantly minority ethnic (96.2%), with 67.7% from poor households. Table 1 presents the main characteristics of the study population by BMI classification. Gender distribution did not differ significantly across BMI categories (*p* = 0.865). However, mean age increased progressively from the thinness to overweight/obese groups (7.63 ± 1.47 vs. 8.18 ± 1.46 vs. 8.60 ± 1.42 years, *p* = 0.048). Most participants belonged to ethnic minority groups (over 90%), with no significant difference across BMI categories (*p* = 0.497).

**Table 1:** Descriptive characteristics of the study population by BMI classification.

Variable	Categories	Thinness (*n* = 140), (%)	Normal (*n* = 623), (%)	Overweight/Obese (*n* = 9), (%)	*p*-value
Gender	Male	48.6	50.4	55.6	0.865
	Female	51.4	49.6	44.4	
Age (years)	Mean ± SD	7.63 ± 1.47	8.18 ± 1.46	8.60 ± 1.42	0.048*
Ethnicity	Kinh	5.3	3.4	11.1	0.497
	Minority	94.7	96.6	88.9	
Household income	Poor	56.3	70.1	77.8	0.011*
	Average or above	43.7	29.9	22.2	
Birth weight (kg)	< 2.5	92.6	94.9	100	0.474
	≥ 2.5	7.4	5.1	0	
Number of siblings	Mean ± SD	1.89 ± 0.32	1.91 ± 0.29	1.89 ± 0.33	0.986

Household income poor/non-poor per Decree 07/2021/NĐ-CP; Data are presented as mean ± SD or %; p-values obtained from Chi-square or ANOVA tests as appropriate; Significant at p < 0.05. No significant differences were found between the two schools in gender or age.

Household income level showed a significant association with BMI status (*p* = 0.011): overweight/obese children were more often from poorer households (77.8%) compared to normal-weight (70.1%) and thin children (56.3%). Birth weight and number of siblings were not significantly associated with BMI.

### Univariate analysis of dietary and lifestyle factors associated with BMI

Table 2 summarizes children’s dietary habits and physical activity according to BMI classification.

**Table 2:** Univariate analysis of dietary habits and physical activity by BMI classification.

Variable	Categories	Thinness (%) (*n* = 140)	Normal (%) (*n* = 623)	Overweight/Obese (%) (*n* = 9)	*p*-value
Meal frequency	< 3 meals/day	14.1	19.7	0	0.134
	≥ 3 meals/day	85.9	80.3	100	
Breakfast consumption	Regular	92.4	95.7	100	0.308
	Irregular	7.6	4.3	0	
Sugary drink consumption	Yes	63.9	71.8	100	0.024*
	No	36.1	28.2	0	
Snack consumption	Yes	82.7	75.2	100	0.057
	No	17.3	24.8	0	
Meat and fat intake	Low	68.0	59.0	66.7	0.166
	Medium	32.0	41.0	33.3	
Physical activity	Regular	22.8	20.5	22.2	0.866
	Irregular	77.2	79.5	77.8	
Screen time (> 2 h/day)	Yes	15.3	22.2	22.2	0.161
	No	84.7	77.8	77.8	

Low meat and fat intake = < 2 servings/week; Medium = 2–4 servings/week; High = > 4 servings/week; Snacks yes: ≥3 times/week processed items like chips, candies.); p-values obtained from Chi-square tests; Significant at *p* < 0.05.

Meal frequency and breakfast consumption were not significantly different between BMI groups (*p* = 0.134 and *p* = 0.308, respectively). However, all overweight/obese children reported consuming at least three meals per day and having breakfast regularly.

Consumption of sugary drinks was significantly higher among overweight/obese children (100%) compared with thin (63.9%) and normal-weight children (71.8%) (*p* = 0.024). Snack consumption approached significance (*p* = 0.057), with all overweight/obese children reporting snack intake.

Meat and fat intake, physical activity, and screen time did not differ significantly by BMI category (*p* > 0.05). Overall, dietary behaviors—particularly sugary drink consumption—appeared to have stronger associations with BMI than physical activity in this population.\.

### Multivariate logistic regression models for thinness and overweight/obesity

Multivariate logistic regression analyses were conducted to identify independent predictors of thinness and overweight/obesity, using normal weight as the reference category (Table 3). All models were adjusted for age (by class) and school, which aligned with reviewer recommendations.

**Table 3:** Multivariate logistic regression models for predictors of thinness and overweight/obesity (reference = normal weight).

Variable	Thinness (aOR, 95% CI)	*p*-value	Overweight/Obesity (aOR, 95% CI)	*p*-value
Gender (Male vs. Female)	1.00 (0.66–1.50)	0.986	1.71 (0.34–8.61)	0.513
Ethnicity (Minority vs. Kinh)	0.59 (0.20–1.75)	0.344	0.19 (0.01–3.40)	0.257
Household income (Poor vs. Average/Above)	0.57 (0.36–0.88)*	0.012	0.84 (0.13–5.40)	0.852
Dieting behavior (Yes vs. No)	0.38 (0.20–0.72)*	0.003	0.59 (0.06–6.14)	0.662
Late-night eating (Yes vs. No)	0.66 (0.39–1.12)	0.127	0.79 (0.13–4.64)	0.792
Soft drink consumption (Yes vs. No)	0.58 (0.36–0.93)*	0.024	-	-
Processed food consumption (Yes vs. No)	1.50 (0.96–2.35)	0.073	2.04 (0.34–12.19)	0.433
Candy/snack consumption (Yes vs. No)	1.89 (1.13–3.19)*	0.016	-	-
Fatty meat consumption (Yes vs. No)	0.61 (0.40–0.94)*	0.026	0.55 (0.11–2.72)	0.464
High screen time (> 2 h/day vs. ≤2 h/day)	0.65 (0.39–1.08)	0.098	0.99 (0.17–5.75)	0.992
Low physical activity (Irregular vs. Regular)	0.83 (0.50–1.37)	0.459	0.98 (0.15–6.25)	0.985

Models adjusted for age (by class) and school; aOR = adjusted odds ratio; CI = confidence interval; Significant at *p* < 0.05.

#### Predictors of thinness

Children from poorer households had significantly lower odds of being in the normal-weight category (aOR = 0.57, 95% CI = 0.36–0.88, *p* = 0.012).

Dieting behavior, defined as self-reported attempts to control weight through food restriction, was also associated with reduced odds of thinness (aOR = 0.38, 95% CI = 0.20–0.72, *p* = 0.003).

Dietary factors showed mixed effects: soft drink consumption (aOR = 0.58, *p* = 0.024) and fatty meat consumption (aOR = 0.61, *p* = 0.026) were negatively associated with thinness, whereas snack consumption was positively associated (aOR = 1.89, *p* = 0.016).

#### Predictors of overweight/obesity

In contrast, none of the examined factors were significantly associated with overweight/obesity.

Wide confidence intervals and non-significant p-values (all > 0.05) suggest the influence of unmeasured determinants such as genetic, familial, or environmental factors on excess weight in this population.

## Discussion

This study highlights the double burden of malnutrition among primary school children in the northern mountainous region of Vietnam, where both undernutrition and overnutrition coexist. The findings align with previous research emphasizing the impact of socioeconomic status, dietary habits, and lifestyle factors on children’s nutritional status^15,16^.

Household income was a significant predictor of thinness, with children from poorer households having lower odds of being in the normal weight category (OR = 0.57, 95% CI: 0.36–0.88, *p* = 0.012). In our study, 56.3% of children classified as thin came from poor households, compared to 70.1% in the regular weight group and 77.8% in the overweight/obese group. This suggests that lower-income children may have reduced access to adequate nutrition, reinforcing findings from Duong et al. (2022) in Vietnam ethnic minorities, where children from low-income backgrounds had a higher prevalence of undernutrition^3^. As in our rural setting, unsafe water and inadequate sanitation significantly contributed to undernutrition.

In addition, the observed progressive increase in age across BMI categories may reflect the cumulative effects of dietary transition with age. Older children are more likely to make independent food choices and have easier access to energy-dense snacks and sugary drinks, which could partially explain their higher BMI values^3,6,17^.

Furthermore, the correlation between household income and BMI classification indicates that economic disparities significantly shape children’s dietary choices and access to nutrient-rich foods. This trend is comparable to findings from Seifu et al. (2024), who demonstrated that children from lower-income families in Ethiopia exhibited higher rates of stunting and wasting due to chronic food insecurity and limited dietary diversity^18^.

Dietary habits play a crucial role in determining nutritional outcomes. In our study, sugary drink consumption was significantly higher among overweight/obese children (100%) compared to normal (71.8%) and thin children (63.9%) (*p* = 0.024). This aligns with findings from Tang et al. (2020) in China, where frequent consumption of sugar-sweetened beverages was strongly linked to childhood obesity^6^. Additionally, our study found that all overweight/obese children consumed snacks, compared to 75.2% of normal-weight and 82.7% of thin children (*p* = 0.057). This supports Aulia et al. (2024), who reported that frequent snacking was associated with increased BMI and poor learning outcomes in Indonesian children^19^.

The significant association between processed food consumption and BMI classification further highlights the impact of dietary behaviors on malnutrition. In our study, the odds ratio for processed food consumption among thin children was 1.5 (95% CI: 0.96–2.35, *p* = 0.073), suggesting that reliance on processed foods may contribute to inadequate nutritional intake. These findings parallel those from Alston et al. (2020), who noted that food insecurity in rural Australia led to increased reliance on calorie-dense, nutrient-poor processed foods, exacerbating malnutrition rates^20^.

Despite global concerns regarding physical inactivity and screen time contributing to childhood obesity, our study did not find significant associations between BMI categories and physical activity levels (*p* = 0.866) or screen time (*p* = 0.161). Regular physical activity was reported by 22.8% of thin children, 20.5% of normal-weight children, and 22.2% of overweight/obese children. This contrasts with findings from Aydin et al. (2021), who observed that inadequate physical activity and excessive screen time were major contributors to childhood obesity in Australian primary schools^8^.

However, our study’s lack of significant associations may be due to the population’s unique geographical and lifestyle factors. Children in the northern mountainous region of Vietnam may engage in higher levels of incidental physical activity related to daily chores and school commutes, mitigating the impact of structured physical activity levels on BMI classification. Similar observations were made by Headey et al. (2018), who found that rural children in Sub-Saharan Africa exhibited lower obesity rates despite limited access to organized physical activity programs^21^.

Our findings are consistent with research from regional trends, which reported high rates of childhood undernutrition in rural areas^22,23^. Similarly, Seifu et al. (2024) identified strong correlations between poverty and undernutrition, further reinforcing our findings that economic status plays a significant role in child nutrition^18^.

Conversely, studies from urbanized regions such as China report rising childhood obesity rates due to dietary shifts toward energy-dense foods and reduced physical activity^17^. In our study, all overweight/obese children consumed at least three meals daily, compared to 80.3% of normal-weight and 85.9% of thin children. This dietary trend suggests an emerging risk of overnutrition in specific segments of rural Vietnamese children, similar to findings from Liu et al. (2024), who documented increasing obesity rates among children in pilot nutrition improvement areas^24^. Comparison with national trends: Aligns with Vietnam’s 16.8% stunting in 5–19 year-olds, higher in minorities^4^.

*Implications for policy and interventions.* These findings emphasize the need for targeted interventions that address undernutrition and overnutrition. School-based nutrition programs have effectively improved child nutrition in low-resource settings (Tran, 2024b)^25^. Expanding access to school meal programs, promoting nutrient-rich local foods, and incorporating nutrition education into school curricula could help mitigate malnutrition in Vietnam’s mountainous regions.

Additionally, targeted policies addressing sugary drink consumption and unhealthy snack intake could help reduce obesity rates. Similar strategies have been successful in China and Australia, where restrictions on sugary beverages in schools have led to declines in childhood obesity rates^8,17^. Furthermore, implementing policies encouraging parental engagement in children’s dietary habits could strengthen long-term nutritional improvements, as Sarma et al. (2015) demonstrated in Sri Lanka^26^.

*Limitations.* This study provides valuable insights into primary school students’ nutritional challenges in a remote region of Vietnam. However, limitations include its cross-sectional design, which prevents causal inferences and potential recall bias in dietary habit reporting. Additionally, unmeasured factors such as genetic predisposition and family eating patterns may influence nutritional outcomes and should be explored in future research. Expanding the sample size and including more diverse geographical regions in future studies could enhance the generalizability of findings; findings may not generalize beyond similar mountainous ethnic areas; the lack of power in the overweight/obesity analysis is discussed.

## Conclusion

The coexistence of undernutrition and overnutrition in the northern mountainous region of Vietnam underscores the urgent need for comprehensive nutritional interventions. Socioeconomic status remains a key determinant of malnutrition, with 56.3% of thin children coming from poor households compared to 70.1% of normal-weight and 77.8% of overweight/obese children. Dietary habits, particularly sugary drink and snack consumption, significantly impact BMI outcomes, with 100% of overweight/obese children consuming sugary drinks and snacks. Future policies should focus on improving food security, enhancing nutritional education, and encouraging balanced dietary practices to promote healthier growth trajectories among children in disadvantaged regions; findings may not generalize beyond similar mountainous ethnic areas.

## Supplementary Information

Below is the link to the electronic supplementary material.

Supplementary Material 1

## Data Availability

The data used in this study are available upon reasonable request from the corresponding author.
