# Supplementary material for: Nutritional status and determinants among primary school students in the challenging terrain of northern mountainous Vietnam
Source: Sci Rep. 2025 Dec 2;15:45793. doi: 10.1038/s41598-025-28827-4 (PMC12756274; doi:10.1038/s41598-025-28827-4)
Supplement: Supplementary file 1 — Supplementary Material 1 [file 41598_2025_28827_MOESM1_ESM.pdf]

Thai Nguyen University **Appendix 1 – Survey Questionnaire**
University of Medicine and Pharmacy

Code of respondent: ……………………..
School code: ……………………..
Date of interview: ……/……/……

Survey on the Nutritional Status of Students at Two Primary Schools in Trung Khanh District, Cao Bang Province

Part 1. General information about the student

Code	Question / Item	Response Options
A1	Full name of student	....
A2	Date of birth	....
A3	Sex	1. Male 2. Female
A4	Ethnicity	1. Kinh 2. Tay 3. Nung 4. Dao 5. Hmong 6. Other (specify): …
A5	Grade/Class	....
A6	Address	....
A7	Height	Measurement 1: …… m Measurement 2: …… m
A8	Weight	Measurement 1: …… kg Measurement 2: …… kg

Part 2. General information about the student's parent (father/mother or primary caregiver)

Code	Question / Item	Response Options
B1	Name of parent	....
B2	Date of birth of parent	....
B3	Highest education level	1. Incomplete primary 2. Primary 3. Lower secondary 4. Upper secondary 5. College/University/Postgraduate 6. Other (specify): …
B4	Occupation	1. Officer/worker 2. Farmer 3. Trader 4. Housewife 5. Other (specify): …
B5	Child's birth weight	....
B6	Number of children in household	....
B7	Birth order of this child	1. First 2. Only child 3. Youngest 4. Other birth order
B8	Average household income per capita per month	....
B9	Is anyone in your family overweight?	1. Yes 2. No 3. Don't know/No answer
B10	If yes, who?	1. Father 2. Mother 3. Sibling 4. Don't know/No answer

Part 3. Parents' knowledge and practices regarding prevention of undernutrition and overweight/obesity

Section C – Knowledge and practices related to undernutrition

Knowledge

Code	Question / Item	Response Options
C1	According to you, which food groups should be included in your child's daily meals? (Multiple choice, order from most to least consumed)	1. Starches (rice, maize, potatoes…) 2. Vegetables and fruits 3. Meat, fish, eggs, soy milk 4. Oil, fat, peanuts, sesame 5. Other
C2	How does malnutrition affect your child's health now and in the future? (Multiple choice)	1. Poor growth 2. Poor intelligence 3. Frequent illness 4. Other (specify) 5. Don't know
C3	When should Oresol (oral rehydration solution) or salted porridge be given?	1. Fever 2. Vomiting 3. Diarrhea
C4	Do you think tooth decay increases risk of malnutrition?	1. Yes 2. No
C5	Do you think worm infection increases risk of malnutrition?	1. Yes 2. No
C6	What are the signs of respiratory infection?	1. Fever 2. Sneezing/runny nose 3. Cough 4. Shortness of breath 5. Other

Practices

Code	Question / Item	Response Options
C7	Do you monitor your child's height and weight?	1. Yes 2. No → skip C9
C8	If yes, how often?	1. Daily 2. Weekly 3. Monthly 4. Never 5. Other
C9	What food groups do you usually give your child?	Same options as C1
C10	How many meals does your child eat per day?	1. One 2. Two 3. Three 4. Four 5. Other
C11	Do you regularly wash hands with soap before/after preparing food and before feeding your child?	1. Always 2. Sometimes 3. Rarely 4. Never 5. Don't know
C12	What do you do when your child refuses to eat?	1. Force eating 2. Change menu 3. Encourage/persuade 4. Other 5. Don't know
C13	How do you adjust meal frequency when your child is ill or has diarrhea?	1. More than usual 2. Same 3. Less 4. Other 5. Don't know
C14	How often do you deworm your child?	1. Never 2. Every 6 months 3. Once a year 4. Other
C15	How do you prevent tooth decay for your child?	1. Limit sweets 2. Brush twice daily 3. Brush once 4. Other 5. Don't know
C16	What do you do when your child has diarrhea?	1. Give Oresol 2. Give salted porridge 3. Digestive enzymes 4. Antibiotics 5. Herbal medicine 6. No treatment 7. Other 8. Don't know
C17	What do you do when your child has acute respiratory infection?	1. Give antibiotics 2. Use herbal medicine 3. Take to health facility 4. Other 5. Don't know

Section D – Knowledge and practices related to overweight and obesity

Knowledge

Code	Question / Item	Response Options
D1	What food groups should be included in daily meals?	Same as C1
D2	What is overweight/obesity?	1. Weight above normal for height 2. Excess fat accumulation 3. A disease affecting health 4. Not much effect 5. Other 6. Don't know
D3	Why do children become overweight/obese? (Multiple choice)	1. Heredity 2. Eating too much rice 3. Eating too much meat 4. Sweets/sugar 5. Fat 6. Fast food 7. Lack of exercise 8. Metabolic disease 9. Other 10. Don't know
D4	How does overweight/obesity affect health? (Multiple choice)	1. Causes hypertension, heart disease 2. Affects bones/joints 3. Reduces memory 4. Lowers academic performance 5. Alters appearance 6. Affects mental health 7. Other 8. Don't know
D5	Does skipping meals relate to overweight/obesity?	1. Yes 2. No 3. Don't know
D6	Does eating more than 3 meals/day relate to overweight/obesity?	1. Yes 2. No 3. Don't know
D7	Do you know the causes of overweight/obesity?	1–7 same as D3 options
D8	How can overweight/obesity be prevented?	1. Skip one meal/day 2. Eat less rice 3. Eat less meat 4. Eat less fat 5. Eat less sugar/sweets 6. Eat more vegetables 7. Balanced, diverse diet 8. Exercise regularly 9. Monitor growth 10. Other 11. Don't know

Practices

Code	Question / Item	Response Options
D9	What food groups do you usually give your child?	Same options as D1
D10	How many meals does your child have per day?	1. One 2. Two 3. Three 4. Four 5. Other 6. Don't know
D11	What do you do when your child eats too much?	1. Change food 2. Maintain normal 3. Reduce food 4. Stop child from eating more 5. Do nothing 6. Other 7. Don't know
D12	Do you often give your child sweets (candy, sugar, cakes, ice cream)?	1. Yes 2. No
D13	Do you often give your child fast food or processed foods (sausages, skewers, etc.)?	1. Yes 2. No
D14	Does your child often eat fruits or drink fruit juice?	1. Yes 2. No

D15	Does your child drink soft drinks/soda regularly?	1. Yes 2. No
D16	Does your child have late-night snacks after 9 p.m.?	1. Yes 2. No

Part 4. Child's eating habits and physical activity (parent interview)

Code	Question / Item	Response Options
E1	How many total meals per day (home + school)?	1–5 options (as above)
E2	Does your child eat breakfast regularly?	1. Yes → times/week 2. No → skip E4
E3	If not, why not?	1. Doesn't like breakfast 2. No preferred foods 3. No time 4. Other
E4	Does your child eat after 9 p.m.?	1. Yes → times/week 2. No
E5	Does your child drink carbonated soft drinks?	1. Yes 2. No → skip E7
E6	If yes, how many cans per week?	1. 1 2. 2 3. 3 4. ≥4 5. None
E7	Does your child eat fast food regularly?	1. Yes → times/week 2. No
E8	Does your child eat fried/stir-fried foods often?	1. Yes → times/week 2. No
E9	Does your child eat sweets often?	1. Yes → times/week 2. No
E10	Does your child eat fatty meats often?	1. Yes → times/week 2. No
E11	Does your child follow any dietary restrictions?	1. Yes 2. No → skip E14
E12	If yes, why?	1. Cultural 2. Weight loss 3. Dislike 4. Peer influence 5. Don't know 6. Other
E13	Which foods are avoided?	(List specific items)
E14	Has your child engaged in physical activity in the past week?	1. Yes 2. No → skip E16
E15	If yes, frequency and duration	… times/week … minutes/day
E16	Does your child watch TV/use Internet/read/play games?	1. Yes 2. No
E17	Total screen time per day (minutes/hours)	....

Enumerator: (Signature, full name)
